# Drug Delivery System in the Treatment of Diabetes Mellitus

**DOI:** 10.3389/fbioe.2020.00880

**Published:** 2020-07-29

**Authors:** Ruichen Zhao, Zhiguo Lu, Jun Yang, Liqun Zhang, Yan Li, Xin Zhang

**Affiliations:** ^1^State Key Laboratory of Biochemical Engineering, Institute of Process Engineering, Chinese Academy of Sciences, Beijing, China; ^2^University of Chinese Academy of Sciences, Beijing, China; ^3^Beijing Chest Hospital, Capital Medical University, Beijing Tuberculosis and Thoracic Tumor Research Institute, Beijing, China

**Keywords:** diabetes mellitus, drug delivery, insulin, gene therapy, nanoparticle

## Abstract

Diabetes mellitus has been described as a chronic endocrine and metabolic disease, which is characterized by hyperglycemia and the coexistence of multiple complications. At present, the drugs widely applied in clinical treatment of diabetes mellitus mainly include insulin, insulin analogs, non-insulin oral hypoglycemic drugs and genetic drugs. Nevertheless, there is still no complete therapy strategy for diabetes mellitus management by far due to the intrinsic deficiencies of drugs and limits in administration routes such as the adverse reactions caused by long-term subcutaneous injection and various challenges in oral administration, such as enzymatic degradation, chemical instability and poor gastrointestinal absorption. Therefore, it is remarkably necessary to develop appropriate delivery systems and explore complete therapy strategies according to the characters of drugs and diabetes mellitus. Delivery systems have been found to be potentially beneficial in many aspects for effective diabetes treatment, such as improving the stability of drugs, overcoming different biological barriers *in vivo* to increase bioavailability, and acting as an intelligent automatized system to mimic endogenous insulin delivery and reduce the risk of hypoglycemia. This review aims to provide an overview related with the research advances, development trend of drug therapy and the application of delivery systems in the treatment diabetes mellitus, which could offer reference for the application of various drugs in the field of diabetes mellitus treatment.

## Introduction

In recent decades, the prevalence of diabetes mellitus has increased globally. According to the 9th edition of Diabetes Atlas by the International Diabetes Federation (IDF), the number of global diabetes patients in 2019 is estimated to be 463 million, which will increase to 578 million by 2030 and 700 million by 2045 (Saeedi et al., 2019). At present, diabetes mellitus has become a kind of serious non-communicable disease that causes high mortality and morbidity rate just next to cardiovascular disease and malignant tumor. As one of the most common chronic diseases, diabetes mellitus is an endocrine and metabolic disease characterized by hyperglycemia and multiple complications. Diabetes mellitus is mainly caused by genetic, environmental influence, microbial infection, immune system dysfunction, and mental factors that result in insufficient insulin secretion and insulin resistance. Patients with diabetes mellitus have long suffered from the devastating complications that could lower their quality of life and threaten their lives. Long-term metabolic disorders will cause multi-system and multi-organ targeted damage and chronic progressive lesions (Wu et al., 2015), such as diabetic retinopathy (Cheung et al., 2010), diabetic nephropathy (Mauricio et al., 2020) and diabetic hypertension (Yamazaki et al., 2018). Additionally, serious acute metabolic disorders will lead to extremes in the spectrum of dysglycemia, such as diabetic ketoacidosis and hyperglycemia hyperosmotic state (Nyenwe and Kitabchi, 2016). Therefore, diabetes mellitus has become a pressing health issue nowadays. According to the standards of the World Health Organization (WHO), diabetes mellitus is classified into type 1 diabetes mellitus (T1DM), type 2 diabetes mellitus (T2DM), gestational diabetes, and special types of diabetes. In China, patients with T2DM account for the majority of all patients, about 90% (Alberti and Zimmet, 1998). This article aims to provide an overview of pathogenesis and treatment of T1DM and T2DM.

T1DM, also known as autoimmune diabetes, is characterized by insulin absolutely deficiency due to the damaged pancreatic β-cell function. Although the etiology of T1DM is not completely understood, the pathogenesis of this disease is thought to be linked with many factors. It’s believed that T1DM is caused by a combination of polygenic and environmental factors. Most of these genetic factors are associated with autoimmunity, such as *HLA*, *PTPN22*, *CTLA-4*, and *IL2RA* (Robertson and Rich, 2018). It has been reported that *HLA* on chromosome 6 is the major genetic risk factors among them (Noble and Valdes, 2011). Otherwise, *INS* polymorphisms are suggested to influence the processes of thymic immune tolerance and protect against T1DM development by regulating the expression and metabolism of insulin (Katsarou et al., 2017). Additionally, immunity has great influence on the T1DM. In patients with T1DM, antigen-presenting cells mistakenly present antigens to helper T-cells, and then produce plenty of specific antibodies against pancreatic β-cells. This process impairs the function of pancreatic β-cells, destroys its ability to synthesize and secrete insulin, which leads to the onset of T1DM. Moreover, it has been found that the oxidative stress plays a critical and pivotal role in the failure of the main glucose regulatory mechanism. As the secretion and action of insulin are controlled by a molecular pathway called as insulin signaling cascade, hyperglycemia-induced oxidative stress could decrease ATP/ADP ratio and disturb the normal Na^+^-K^+^ ratio, which causes a depolarization of the membrane and more influx of Ca^2+^, accelerates the decomposition of membrane phospholipids and damages pancreatic cells (Newsholme et al., 2016). Besides, the apoptosis of pancreatic β-cells in T1DM patients is mainly related to the apoptotic pathways formed by three kinds of cytokines, including IL-β, TNF-α, and IFN-γ. Among them, IL-β can inhibit the normal physiological function of pancreatic β-cells, while TNF-α and IFN-γ can synergistically enhance the cytotoxicity of IL-β (Kaminitz et al., 2017). Therefore, the treatment of T1DM should focus on reconstructing the immune tolerance of pancreatic β-cell and protecting the function of it.

**FIGURE 1:**
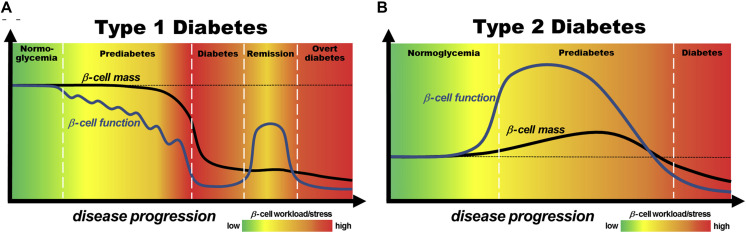
Models of the contribution of pancreatic β-cell mass and function to pathogenesis of T1DM **(A)** and T2DM **(B)**. Reprinted from a previous study Chen et al. (2017) with permission.

As a kind of non-insulin dependent diabetes, the pathogenesis of T2DM is mainly including insulin resistance, impaired pancreatic β-cells function, obesity, oxidative stress (Ighodaro, 2018) and genetic susceptibility. Among them, it is considered that insulin resistance and impaired pancreatic β-cell function are the primary pathophysiological changes of T2DM. Due to many factors of abnormal metabolic process *in vivo*, the efficiency of insulin-mediated glucose uptake and utilization by skeletal muscle, adipocyte and liver decreases. In order to maintain normal blood glucose levels, pancreatic β-cells then compensate for excessive insulin secretion, which results in hyperinsulinemia. The excessive concentration of insulin in plasma causes less sensitivity of target cells to it, which leads to the depletion of pancreatic β-cells and insufficient synthesis and secretion of insulin (Odegaard and Chawla, 2013). In addition, when nutrients are ingested than needed, the excess nutrients are mainly stored as fat in the adipocytes. With storing fat and increasing adipocytes size, it has been noted that the cells seem to be suppressed to uptake glucose and synthesis muscle glycogen, and the hepatic glucose output is excessive. These changes further aggravate insulin resistance that is described as the main cause of T2DM (Hotamisligil, 2006). Therefore, the treatment of T2DM should focus on increasing the sensitivity of target cells to insulin and protecting pancreatic β-cell. Moreover, according to the pathogenesis of T1DM and T2DM, it is shown that β-cell plays a significant role in the onset of diabetes mellitus and we could see the contribution of β-cell mass and function to pathogenesis of disease in Figure 1.

## Drug Therapy

As the pathogenesis of diabetes mellitus is relatively complicated, the crux to control and therapy diabetes mellitus is combined with the patients’ individual circumstances to make self-management including diet modification, appropriate exercise, glucose close monitoring, mood assessment, and drug treatment in combination. For drug treatment, the anti-diabetic drugs are mainly consisting of insulin, insulin analogs and non-insulin hypoglycemic drugs which contain insulin sensitizers, insulin secretagogues and glucose regulators and gene therapy. This article reviews the recent perspectives about common anti-diabetic drugs.

### Insulin and Insulin Analogs

In terms of the absolute or relative deficiency of insulin secretion in patients with T1DM and severe T2DM, insulin is one of the main and indispensable exogenous drugs in the treatment of diabetes. The main physiological functions of insulin and its analogs are to regulate the metabolism of sugar, fat and protein in vivo and maintain blood glucose levels in the normal range. They can transport glucose in plasma into cells by stimulating the target cell membrane carriers in muscle and adipose tissue, accelerate glycogen synthesis in liver and muscle cells, and inhibit glycogen decomposition and the synthesis of PEP carboxykinase (Moroder and Musiol, 2017).

Since insulin was discovered in 1922, it has experienced the development process from animal insulin, recombinant human insulin to insulin analogs (Tibaldi, 2014). But as a polypeptide compound, several disadvantages such as poor stability and rapid metabolism in vivo limited the application of human insulin in clinic. Hence, with the further understanding of molecular structure and composition of insulin, human insulin analogs were synthesized by genetic engineering technology with the amino acid sequence and structure locally modified. As the physical, chemical and pharmacokinetic characteristics of insulin analog are changed, it can simulate the metabolic process of endogenous insulin in vivo more accurately and meet the physiological needs of humans. According to the pharmacodynamic time, insulin analogs can be divided into three categories: rapid-acting analogs, long-acting analogs, and premixed insulin analogs.

Compared with human soluble insulin, the rapid-acting analogs were developed to accelerate insulin absorption, and simultaneously minimize postprandial glucose rise more effectively and lower the risk of hypoglycemia due to the high exogenous insulin concentrations for longer needed (Senior and Hramiak, 2019). Three rapid-acting analogs are currently available, including insulin lispro (Humalog), insulin glulisine (Apidra), and insulin aspart (NovoLog). These insulin analogs are primarily suitable for patients with postprandial hyperglycemia, which can be injected 0–15 min before or immediately after meal, and always with great compliance.

Composed of quick-acting insulin analogs and protamine-crystallized insulin analogs in an appropriate proportion, premix insulins were designed to effectively control the fluctuation of blood glucose after 2 h of the meal and meet the daily needs of basic insulin supplement through regulating the level of fasting blood glucose (El Naggar and Kalra, 2017). Moreover, they could also maximize patient convenience and reduce the number of daily injections. Premixed aspartate insulin 30, premixed aspartate insulin 50 and premixed lysine insulin 25 are commonly applied in clinical treatments. Doctors should flexibly adjust the ratio and dosage of antidiabetic drugs according to the patient’s various conditions to obtain the best pesticide effect.

There are three main treatment strategies of insulin and its analogs, including supplement therapy, replacement therapy, intensification therapy (Cichocka et al., 2016).

For patients with supplementary therapy, they are usually suggested to inject intermediate-acting insulin or long-acting insulin analogs before bedtime to inhibit the output of liver sugar and control fasting blood glucose levels. About 6–8 h after injection, the hypoglycemic effect peaks could effectively combat the “dawn phenomenon.” For some patients who may have poor blood sugar control after dinner, they can choose to increase the injection before breakfast to ensure insulin concentration after dinner. At the same time, this treatment should be attached great attention to the possible occurrence of hypoglycemia at night. Supplementary therapy is always appropriate for patients whose pancreas function has not been completely lost. On the basis of reasonable diet and appropriate exercise, oral hypoglycemic drugs combined with basic insulin are used to maintain blood glucose homeostasis. This treatment can not only regulate and control the blood glucose level, but also alleviate the pancreatic cells burden and protect the pancreatic function by supplementing appropriate amount of exogenous insulin. At the same time, it also has the advantages of low insulin dosage, reducing the incidence of weight gain, and with high compliance.

The patients suitable for insulin replacement therapy are mainly consisting of T1DM whose pancreatic function is severely damaged with absolute loss of insulin secretion, and T2DM patients with a long duration of disease who are insensitive to oral hypoglycemic drugs with the liver and kidney hypofunction. At present, the insulin replacement therapy is widely applied to clinic. There are always two alternative projects for patients, one is injecting premixed insulin twice a day before breakfast and dinner or three times a day before or immediately after a meal, and another is injecting short-acting insulin before meals combined with basic insulin injection before bedtime.

Intensification therapy of insulin refers to daily multiple injections of insulin or using an insulin pump to simulate insulin secretion under physiological conditions to control blood glucose to reach normal level. Intensive treatment is applicable to patients with T1DM, newly diagnosed T2DM with HbA1c > 9%, T2DM with sudden deterioration and gestational diabetes. This treatment can not only control the glucose concentration, but also lessen the further damage of lipotoxicity and glucotoxicity to pancreatic β-cells function.

### Non-insulin Hypoglycemic Agents

Non-insulin hypoglycemic drugs are first-line agents for patients who struggle for maintaining normal blood glucose levels just through diet adjustment and moderate exercise. Currently, there are wide varieties of these drugs commercially such as biguanide, sulfonylurea, thiazolidinedione and glinide. Additionally, there are many newly developing drugs including dipeptidyl peptidase-4 (DPP-4) inhibitors, glucagon-like peptide-1 receptor agonists and sodium-glucose cotransport protein 2 inhibitors. Furthermore, more attention was focused to the free fatty acid receptor 1 agonists, glucokinase agonists and protein tyrosine phosphatase-1B inhibitors, which are still under research. According to the action mechanism of drugs, they could be divided into insulin sensitizers, insulin secretagogues, glucose regulators.

#### Insulin Sensitizers

Adenosine 5′-monophosphate activated protein kinase (AMPK) is a serine/threonine kinase that is ubiquitously expressed in various tissues and cells, such as brain, heart, liver and skeletal muscles. As an intracellular fuel-sensing enzyme, it is involved in bonding the energy sensing to the metabolic manipulation and also contributes to better energy balance in cells (Coughlan et al., 2014). Research shows that AMPK activated by correlative upstream kinases is able to promote the glucose uptake and the oxidative metabolism of lipids in skeletal muscles and liver, and suppress the glycogenesis in liver and lipid synthesis. It has a strong effect on cell energy metabolism and ameliorates insulin resistance (Zhang et al., 2009). Additionally, it also plays a crucial role in controlling many other physiological actions such as cell growth and proliferation, mitochondrial function and biogenesis. Moreover, it also modulates physiological events via the phosphorylation of key enzymes and transcriptional activators which are associated with insulin resistance, such as inflammation, oxidative and endoplasmic reticulum stress (Garcia and Shaw, 2017). Considering its pivotal role in controlling energy homeostasis, AMPK has attracted widespread attention as a potential therapeutic target for metabolic diseases, especially for T2DM. In recent years, AMPK direct activators under the research mainly include Imeglimin, O-304 and KU-5039. However, due to the frequent expression of AMPK *in vivo*, AMPK activation in the heart and brain may have potential side effects. Therefore, how to enhance the specificity and selectivity of these drugs may become the hotspot of future research.

Because protein phosphorylation-dephosphorylation is quite fundamental and versatile mechanism for the control of cellular functions, aberrant tyrosine phosphorylation is linked with the development of many diseases. As a primary non-transmembrane phosphotyrosine phosphatase in various tissues and cells, protein tyrosine phosphatase 1B (PTP 1B) is considered as indispensable part of multiple physiological processes such as regulating cell growth and differentiation, gene transcription, intercellular signal transduction, and immune response (Owen et al., 2013). Currently, PTP 1B has been described as a promising therapeutic target in the effective management of diabetes. The effect of PTP 1B on blood glucose level is mainly related to pancreatic β-cells, leptin signal transduction, and endoplasmic reticulum stress. When the blood glucose concentration increases, it stimulates pancreatic β-cells to secrete insulin, which acts on muscles, liver, fat and other organs to regulate glucose transport, glycogen synthesis and other processes to control blood glucose level in the normal range. Meanwhile, it also acts on pancreatic β-cells itself, promotes its proliferation and differentiation and inhibits apoptosis. However, PTP 1B causes negative regulation of insulin signal transduction and inhibits pancreatic β-cell proliferation (Johnson et al., 2002). In addition, PTP 1B also affects apoptosis by regulating cytokines related to pancreatic cell apoptosis.

However, PTP 1B will cause dephosphorylation and inactivation of leptin-activated JAK2, which in turn affects leptin signal transduction (Qian et al., 2016). Endoplasmic reticulum stress could result in obesity-related insulin resistance. Studies have shown that PTP1B is distributed on the endoplasmic reticulum membrane and endoplasmic reticulum stress up-regulates the expression of PTP1B, which in turn inhibits glucose uptake (Panzhinskiy et al., 2013). Therefore, PTP 1B inhibitors would enhance insulin sensitivity by blocking the PTP 1B-mediated negative insulin signaling pathway and maintain euglycemia (Abdelsalam et al., 2019). However, due to the research issues about cell membrane permeability and selectivity, there are not many PTP-1B inhibitors currently in research stage, except TTP-814 and ISIS-PTP1BRx.

#### Insulin Secretagogues

Free fatty acid receptor-1 (FFAR-1), also known as G protein-coupled receptor 40 (GPR40), belongs to the family of G protein-coupled receptors, which is encoded by the FFAR-1 genes in humans. It has been noted that FFAR1 is strongly expressed in pancreatic β-cells and enteroendocrine cells of the gastrointestinal tract. When blood glucose levels increase, intracellular glucose metabolism accelerates, depolarizing the membrane and closing the ATP-dependent potassium channel (KATP). The voltage-dependent Ca^2+^ channel is then opened. Subsequently, the binding of free fatty acids to GPR40 promotes extracellular Ca^2+^ influx by the phosphatidylinositol signal transduction pathway, which further increases the intracellular Ca^2+^concentration, and then stimulates glucose-dependent insulin secretion (Tanaka et al., 2014). As previously reported, FFAR-1 could not only directly stimulate insulin secretion from pancreatic β-cells, but also act on the enteroendocrine cells of the gastrointestinal tract. Its activation stimulates incretins secretion, activates GLP-1 receptors and indirectly promotes insulin secretion (Christiansen et al., 2013). The glucose-dependent secretion of insulin reduces the probability of hypoglycemia, which makes GPR40 an excellent target for developing therapies that could be efficacious with fewer side effects. At present, the agonists of GPR40 are mainly divided into four categories: thiazolyl derivatives, phenoxyacetamide derivatives, propionic acid derivatives, and pyrrolyl analogs. It has reported that the drugs under the research are including JTT-851 in clinical Phase II, P-11187 and LY-2881835, which are all in clinical Phase I (Poitout and Lin, 2013).

**FIGURE 2:**
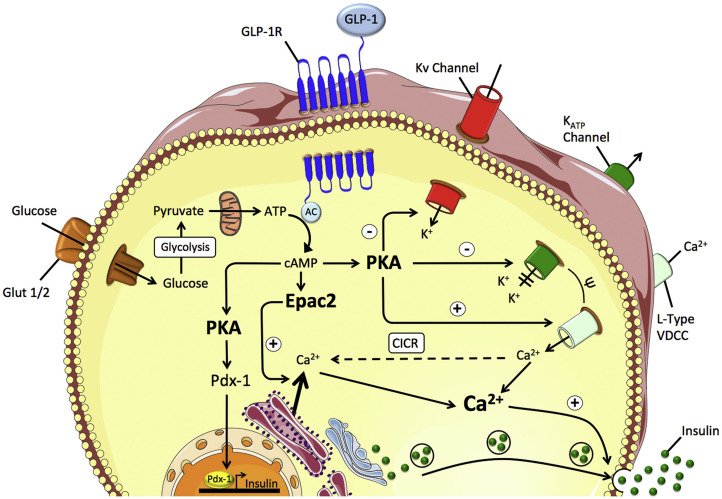
GLP-1 mediated insulin secretion in the pancreatic β-cell. Reprinted from a previous study Müller et al. (2019) with permission.

Glucagon-like peptide-1 (GLP-1) is an endogenous glucagon that is encoded by the proglucagon gene and secreted by intestinal L cells in the colon and rectum. As shown in Figure 2, GLP-1 acts through multiple mechanisms to treat diabetes mellitus. GLP-1 binds to the receptor and directly acts on pancreatic β-cells to promote glucose concentration-dependent insulin synthesis and secretion (Klinger et al., 2008). GLP-1 can inhibit the apoptosis of pancreatic β-cells, stimulate pancreatic ductal cells to differentiate into pancreatic β-cells, and promote the proliferation and differentiation of pancreatic β-cells (Piro et al., 2014). GLP-1 also inhibits the glucagon secretion of pancreatic β-cells. Additionally, by binding to receptors distributed in the gastrointestinal tract, GLP-1 inhibits gastrointestinal peristalsis, reduces nutrient absorption and uptake, and also acts on the central nervous system to suppress appetite (Ji, 2017). However, given natural GLP-1 is unstable *in vivo*, and is easily degraded and inactivated by dipeptidyl peptidase, GLP-1 receptor agonists come into being, which could better meet the clinical needs. Due to a combination of multiple pathways and glucose-dependent insulin secretion, GLP-1 receptor agonist exhibits a stable hypoglycemic effect which reduces the probability of hypoglycemic reaction and also displays many superiorities such as losing weight, reducing blood pressure, regulating blood lipids, and playing a protective role on cardiovascular and kidney. At present, GLP-1 receptor agonists widely used in clinical treatment are mostly injection, and the common ones include short-acting preparations liraglutide, benalutide, and long-acting preparations abilutide, somalutide, etc. (Gentilella et al., 2019).

Dipeptidyl peptidase-4, an enzyme, which could cleave and inactivate many regulatory peptides containing the CD26 target sequence such as GLP-1. Secreted from the L cells of the intestines finishing meals, GLP-1 acts on increasing glucose-dependent insulin secretion from β-cells and inhibiting glucagon release. Additionally, it could also retard gastric emptying and promote satiety by function of the brain (Wang et al., 2018). The predominant glucose-lowering impact of DPP-4 inhibitors is mediated by inhibiting the activity of DPP-4. DPP-4 inhibitors reduce the plasma activity of DPP-4 by 70–90% and increase the levels of circulating GLP-1 by approximately fourfold to strengthen its hypoglycemic effects. At the same time, DPP-4 inhibitors also have the advantages of reducing the risk of hypoglycemia, reducing patient weight, reducing oxidative stress, improving inflammation, and protecting renal function (Davis et al., 2019). DPP-4 inhibitors constitute a novel class of hypoglycemic agents confirmed to improve glycemic control and preserve β-cells function, which have gained widespread use in diabetes treatment. The commercial ones mainly consist of viglitine, aglitine, liglitine, saglitine, siglitine, etc. Moreover, DPP-4 inhibitors are usually recommended in combination with metformin, sulfonylurea, thiane diketone or basal insulin rather than monotherapy. However, for patients who are intolerant to metformin, monotherapy could be the better choice.

#### Glucose Regulators

Sodium-glucose cotransporter 2 (SGLT2) inhibitor has been proposed as a novel class of hypoglycemic drugs that is independent on the pathway of promoting insulin secretion. Sodium–glucose cotransporters 1 (SGLT1) and SGLT2 are indispensable mediators of epithelial glucose transport. While SGLT1 accounts for most of the dietary glucose uptake in the intestine, SGLT2 is responsible for the majority of glucose reuptake in the tubular system of the kidney. Under normoglycemic conditions, about 180 g of glucose is filtered from the original urine every day and then almost all of it is reabsorbed by the proximal tubules, of which about 97% is mediated by SGLT2 and 3% is mediated by SGLT 1 (Bonner et al., 2015). But for patients with diabetes who have already suffered from hyperglycemia, the enhancement of glucose reabsorption in the renal tubules would make the blood glucose concentration for much worse. Therefore, the use of SGLT2 inhibitors competitively bind glucose with transporters, inhibit renal tubular reabsorption of glucose, and assist excess glucose to be excreted with urine to regain euglycemia. At the same time, the inhibitors do not act on pancreatic cells or intestinal cells to aggravate the burden of insulin secretion, which plays a protective role in the function of pancreatic β-cells. In addition to having a good hypoglycemic effect, SGLT-2 inhibitors also have the function of protecting cardiovascular and kidney and lowering blood pressure, lipid and uric acid (Nespoux and Vallon, 2018). However, through the results of clinical trials, SGLT-2 inhibitors also have some adverse reactions, mainly including ketoacidosis, hypoglycemia and urogenital system infection. At present, the listed SGLT-2 inhibitors include daglitazone, englenet, ruglietin, caglione, eglitoglione, etc. (Wanner and Marx, 2018).

11β-hydroxysteroid dehydrogenase (11β-HSD), a NADP(H)-dependent enzyme catalyzing the conversion between bioactive and inert glucocorticoids, is divided into two subtypes, 11β-HSD1 and 11β-HSD2. As a class of steroid hormones secreted by the adrenal tract, glucocorticoids play a significant role in controlling physiologic homeostasis. When present in excess, it could antagonize the role of insulin and reduce the sensitivity of tissues and organs to insulin, which causes insulin resistance. And it could also reduce the cellular uptake of glucose and promote gluconeogenesis, which would lead to abnormal blood glucose levels. At the same time, it has a detrimental impact on blood pressure and lipid level (Chapman et al., 2013). At the same time, 11β-HSD1, highly expressed in important metabolic tissues, such as liver, pancreas, skeletal muscle and fat, can convert cortisone to cortisol and amplify glucocorticoid action locally in a tissue specific manner. This in turn induces a variety of glucocorticoid-mediated reactions, including inhibition of glucose ingestion and promoting gluconeogenesis (Hollis and Huber, 2011). Therefore, 11β-HSD1 inhibitors are used to reduce the activation of glucocorticoids, enhance the insulin sensitivity of related tissues and organs, inhibit gluconeogenesis, and then regain normal blood-glucose levels. In clinical trials, 11β-HSD1 inhibitors have been well tolerated and have improved glycemic control, lipid profile and blood pressure, and induced modest weight loss. Therefore, the drugs including VTP-34072, HIS-388, EQ-1280, CNX-010 and so on are under development, which could be great choices for diabetes treatments.

### Gene Therapy

Currently, it has been found that the treatments of non-insulin hypoglycemic drugs, or insulin and its analogs can only temporarily minimize the symptoms of hypoglycemia, but could not permanently improve the function of islet cells, maintain blood glucose homeostasis, and avoid various complications. Additionally, it is impossible to administer exogenous insulin to produce an insulin profile that exactly mimics the natural dynamics of insulin. And the cycle pathway of insulin injection systemically is quite different from the one taken by insulin secreted from the endocrine pancreatic β-cells. Gene therapy refers to transfer exogenous genes into appropriate recipient cells in patients to prevent or cure a particular disease (Yan et al., 2018). Gene therapy is a promising strategy for the treatment of diabetes mellitus as it actually targets the root cause of diseases and enables us to arrest or reverse a condition. The main genetic drugs used in gene therapy include DNA, small interfering RNA (siRNA), mRNA, microRNA or antisense oligonucleotides. The gene therapy for diabetes mellitus could be divided into replacement gene therapy, immune gene therapy and regulatory gene therapy.

#### Replacement Gene Therapy

**FIGURE 3:**
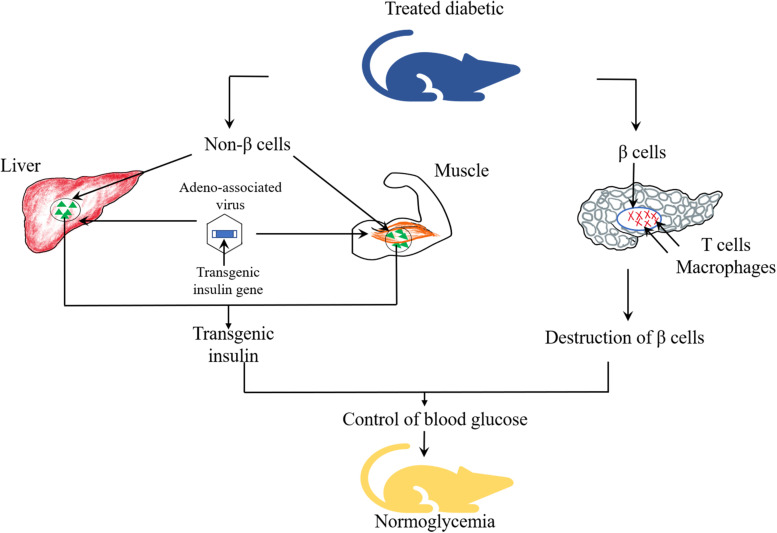
Replacement gene therapy by production of therapeutic transgenic insulin for T1DM.

Given that different degrees of damage has been found in the pancreatic cells of patients with T1DM and T2DM, as shown in Figure 3, non β-cells are capable of secreting insulin, which can be constructed to replace the damaged pancreatic β-cells to play a role in remedying and redressing the deficiency of insulin synthesis and secretion. Successful replacement gene therapy should satisfy several important conditions (Yoon and Jun, 2002): (1) an effective insulin gene transfer system; (2) a regulatory system with response to glucose to control the expression and release of insulin; (3) the transfected cells are capable of processing proinsulin into mature and active insulin; (4) target cells with biochemical properties similar to β-cells but not be attacked by the immune systems.

Viral vectors such as lentivirus and adeno-associated virus, and non-viral vectors such as liposomes and plasmids have been utilized to deliver genes into target tissues or cells, such as pancreas, liver, intestinal endocrine K cells and muscle cells. Among them, intestinal endocrine K cells, which have many similarities with pancreatic β-cells, could produce glucose-dependent insulinotropic polypeptide (GIP) and contain prohormone converting enzymes essential for proinsulin processing (Ahmad et al., 2012). Studies demonstrated that transgenic mice induced by streptozotocin (STZ), after transferring the GIP promoter into K cells of the gastrointestinal tract area, showed long term euglycemia (Tudurí et al., 2012). These results indicated that K cells could produce sufficient amount of insulin to maintain glucose homeostasis. Romer and Sussel (2015) introduced adeno-associated viral vectors carrying insulin and glucokinase genes into the skeletal muscle of STZ-induced diabetic mice and dogs. The co-expression of these two genes enhanced the translocation of GLUT4 and glucokinase, and elevated glucose transport into muscle cells. In addition, glucokinase could act as “glucose sensor” to regulate the secretion of insulin according to the changes in blood glucose concentration. Xiao et al. (2018) infused adeno-associated viruses as vectors to carry Pdx1 and MafA expression cassettes to reprogram α-cells into functional β-cells to restore the damaged β-cell function. As a potentially ideal source of β-cells replacement, α-cells possess the following advantages. As endocrine cells, the growth process of α-cells is similar to that of β cells, which may be beneficial for reprogramming (Bramswig and Kaestner, 2011). Particularly, a large percentage of α-cells are found in human islets, which constitute a potentially abundant source for reprogramming (Bramswig and Kaestner, 2010). Appropriate reduction in the number of α-cells does not affect the normal glucose metabolism and is conducive to control the blood glucose (Shiota et al., 2013). Furthermore, it is suggested that the extreme loss of β-cells may be the trigger of α-to-β cell conversion (Thorel et al., 2010). Lastly, ATAC sequencing (ATAC-seq) studies have shown that α-cell genomes are notably accessible and easier to transdifferentiate (Ackermann et al., 2016). At the same time, the results of this study revealed that normal glucose level was observed in alloxan (ALX)-induced diabetic mice for 4 months, and it was also found that new INS+ cells were almost derived from α-cells. The results proposed that α-cells may be the ideal target cells in replacement gene therapy.

#### Immune Gene Therapy

Immune gene therapy is generally applied to patients with early T1DM. In view of the complicated autoimmune mechanism of T1DM involving diversified cells and multiple signaling pathways, current researches attempt to block or reverse the process of autoimmune response by transducing target genes. The immunological intervention might be able to protect the function of islet cells and reduce the reliance of the patient on insulin administration.

IL-10, an anti-inflammatory cytokine with multidirectional biological activities, possesses the function to change the immune response of the organism and the expression of MHC class II antigens. It could also mediate the mutual regulations between Th1 and Th2 cells and have suppressive effect in preventing autoimmune disease (Xu et al., 2015). It has been reported that intramuscular recombinant adeno-associated viral vector encoding murine IL-10 (rAAVIL-10) was injected into non-obese diabetic mice. Among them, 60% of the non-obese diabetic mice receiving high-dose of rAAV-IL-10 maintained euglycemia for at least 117 days, while diabetes mellitus recrudesced within 17 days in those mice which received a low-dose of rAAV-IL-10 (Zhang et al., 2003). The high level of IL-10 expression had a positive effect on the reduction of autoimmunity. The ability to alter antigen specificity of T cell receptor (TCR) or chimeric antigen receptor (CAR) gene transfer promotes personalized cellular immunotherapy for cancer. In contrast, this method can reduce inflammation by changing the specificity of regulatory T cells (Tregs) in the autoimmune environment (Ehlers, 2016). Howard et al. (Yeh et al., 2017) designed an efficient protocol, lentiviral gene transfer of TCRs, which recognizes T1DM-associated autoantigens to achieve tissue-specific induction of antigen-specific tolerance and prevent β-cell destruction. Hereby, it has been shown that rapid amplification of antigen-specific Tregs was feasible to alleviate β-cell autoimmunity. Meanwhile, a study conducted by Coleman et al. (2016) demonstrated that immune precursor cell-mediated gene therapy mitigated the destruction of pancreas and restored long-term tolerance of islet antigens by terminating the response established of antigen-specific memory T cells. Therefore, gene therapy mediated by immune precursor cells may be one of the interventions for immunotherapy against T1DM.

#### Regulatory Gene Therapy

In terms of the generation and maturation of pancreatic β-cells, and the synthesis and secretion of insulin, there are dozens of cytokines involved in regulation. The expression of various genes and the activation and inactivation of diversified proteins are regulated with a set of precise procedures. Therefore, the researchers attempted to transfer the genes encoding interrelated cytokines into the organism to facilitate the normal secretion of insulin and maintain blood glucose homeostasis.

Insulin-like growth factor 1 (IGF1) is a β-cell mitogen and pro-survival factor which could enhance the absorption of glucose and amino acids, promote the synthesis of glycogen, and improve the sensitivity of organs to insulin. In addition, IGF1 regulates immune functions and is one of the main participants in the crosstalk between immune and endocrine system (Smith, 2010). It has been found that IGF1 overexpressing in β-cells arrested the overexpression of human interferon-β (IFN-β) in β-cells, prevented the islet infiltration and immune cell-mediated β-cell death in transgenic mice (Casellas et al., 2006). In a study conducted by Mallol et al. (2017), AAV of serotype 8 (AAV8-IGF1-dmiRT) encoding IGF-1 was constructed and injected into the pancreatic alveolar cells of adult mice, while using microRNA target sequences to achieve tissue-specific gene expression. The results showed that the expression of IGF1 in pancreas could prevent the onset of diabetes in non-obese mice by blocking β-cell-directed autoimmune attack. Therefore, AAV-mediated IGF-1 gene transferring with microRNA has great therapeutic potential for T1DM treatment and prevention.

### The Challenges of Drug Therapy Faced

Though various antidiabetic drugs are flooding into the market and widely applied into diabetes management, complete and successful cure of diabetes mellitus still remain untouched because of several intrinsic deficiencies and adverse effects of these drugs revealed. The optimal drug concentration needed couldn’t reached in focal areas due to the chemical instability and sensibility to proteolytic degradation of drugs in harsh physiological environment. Moreover, the conventional dosage forms can’t be intelligently adjusted according to the wide fluctuation in glucose concentration, which results in high risk of hypoglycemia. The drugs could not also accumulate into the desired site, which might cause severe side-effects on other organs. In addition, other challenges of drugs therapy faced, such as difficulties in effective absorption and uptake by the target cells, shorter plasma half-life time, narrow therapeutic window, low bioavailability and poor patient compliance, also should be settled urgently.

## Application of Drug Delivery Systems in Diabetes Mellitus Treatments

Due to the challenges of pharmacological therapy faced and the superiorities of nanoparticles (NPs) in drug delivery and imaging (Rai et al., 2016), researches have put increasing interest in nano carriers in the treatment and management of diabetes mellitus. The composition of systems for drug delivery mainly includes liposome, polymer-based NPs, and inorganic NPs. Among them, diverse polymer-based NPs including nanospheres, nanocapsules, micelles, and dendrimers are developed as suitable drug carriers. Table 1 contains several types of nano carriers used for loading insulin and other antidiabetic drugs, and summarizes their reported effects *in vivo*. These nano carriers have been found to be potentially beneficial in many aspects, such as protecting drugs from enzymatic degradation, improving their stability, overcoming different biological barriers *in vivo*, and increasing bioavailability. They could also act as an intelligent automatized system to mimic endogenous insulin delivery and possess a non-linear response to an external signal, which reduces the risk of hypoglycemia and obtain better compliance of patients. Moreover, they have great performance in more precisely delivering drugs to the targeted sites and sustaining and controlled release of drugs within targeted sites over a long period, which could minimize the undesirable side-effects and maximize the therapeutic effect (Wang J. Q. et al., 2019). Otherwise, quantum dots and metal-oxide NPs are widely applied to the detection of pH and chemical analytes and imaging in drug delivery because of their unique photoluminescent properties. At the same time, the properties of polymer materials, the mean particle size and polydispersity, the surface electrical charge and hydrophilicity of nanoparticles are crucial for the delivery of antidiabetic drugs (Souto et al., 2019). Therefore, it is quite necessary and significant to develop appropriate NP delivery systems for effective diabetes treatment.

**TABLE 1:** Delivery systems applied for the treatment of diabetes mellitus.

Type of delivery system	Drug	Administration route	Effects *in vivo*	References
Liposomes	Complexes of Cas9-RNP	Subcutaneous	Alleviate insulin resistance and the damage of liver and kidney	Cho et al., 2019
Liposomes	Bovine serum albumin and insulin	Oral	Conquer the mucus and epithelium barriers	Wang A. H. et al., 2019
Dextran nanoparticles	Insulin	Subcutaneous	Prolonged hypoglycemic effect	Gu et al., 2013a
Polyethylene glycol (PEG) nanoparticles	Insulin	Oral	Enhance hypoglycemic effects	Wu et al., 2017
			Improve bioavailability	
PLGA nanoparticles	Insulin	Oral	Prolonged hypoglycemic effect	Sheng et al., 2015
Chitosan nanoparticles	Insulin	Oral	Overcome the mucus and epithelium barriers	Wong et al., 2017
			Enhance bioavailability	
SiO**_2_** nanoparticles	Metformin	Transdermal	Enhance hypoglycemic effect	Zhang et al., 2018
			Lower risk of hypoglycemia	
Hydrogels	Insulin	Oral	Targeted delivery	Wood et al., 2008
			Enhance bioavailability	
Dendrimers	Human and bovine pancreatic insulin Calcitonin	Subcutaneous	Enhance glucoregulatory effects	Kesharwani et al., 2018
Micelles	Lyophilized human and porcine insulin	Oral	Prevention of aggregation of insulin	Li X. H. et al., 2015
			Enhance bioavailability	
				

### Nanoliposome

Liposomes are described as spherical vesicles composed of one or more lipid bilayers, which are formed by the self-assembly of phospholipids. Both hydrophilic and hydrophobic drugs with low permeability could be encapsulated either in hydrophilic interior aqueous core or hydrophobic lipid bilayers, or even be bound to the surface of the vesicle (Wong et al., 2018a, b). The advantages of great biocompatibility, biodegradability, poor-immunogenicity, protective effect against enzymatic degradation and cell-specific targeting make liposomes attractive vehicles in the field of drug delivery.

**FIGURE 4:**
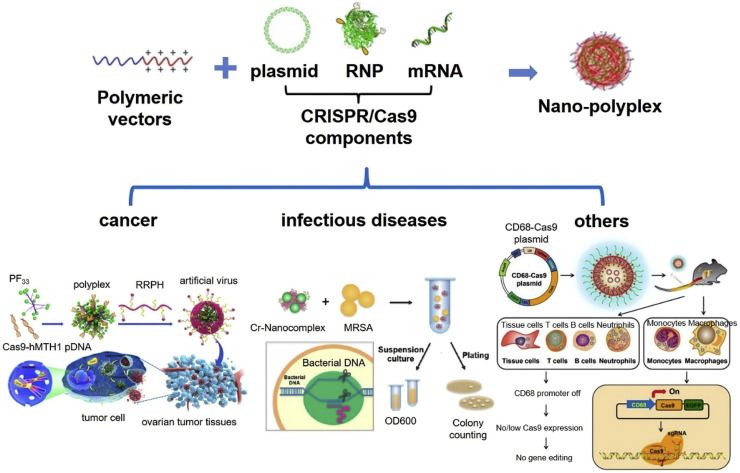
Cationic polymeric nanoformulation and their application in CRISPR/Cas9 system delivery for gene therapy. Reprinted from a previous study Chen K. F. et al. (2019) with permission.

As shown in Figure 4, in the CRISPR/Cas9 system, cationized Cas9 proteins and a single guide RNA (sgRNA) constitute a highly anionized RNP (ribonucleoprotein) complex (Gupta et al., 2019). For better cell membrane permeability and protein stability, the complex could be encapsulated with cationic liposomes and then delivered to cells by endocytosis and macropinocytosis. As positively charged lipid vesicles, cationic liposomes interact with negatively charged gene therapy drugs that are effectively compressed from extended structures to smaller particles through electrostatic interaction to form a transfection complex (Song, 2017). Lecithin liposome was designed as a nano carrier to encapsulate complexes of Cas9-RNP through polymer fusion self-assembly for target delivery to liver. In this delivery system, the sgRNA was specifically optimized for dipeptidyl peptidase-4 gene (DPP-4) to inhibit the degradation of glucagon-like peptide 1 to enhance the secretion of insulin (Cho et al., 2019). Results showed that T2DM mice injected with nano carrier Cas9-RNP complexes exhibited the remarkably down-regulation of DPP-4 gene, accompanied by euglycemia, insulin response, and alleviated liver and kidney damage. These results suggest that the nano-liposomal carrier system with therapeutic Cas9-RNP had great potential for the treatment of T2DM.

Wang A. H. et al. (2019) designed a delivery system to conquer the mucus and epithelium barriers and improve the oral bioavailability of insulin. In this system, bovine serum albumin (BSA) is adsorbed to cationic liposomes (CLs) loaded with insulin to form protein corona liposomes (PcCLs). Further study of the behavior of PcCLs suggests that BSA corona could be shed from PcCLs when they cross the mucus layer, which leads to the exposure of CLs to enhance the transepithelial transport. And investigation shows that, the uptake amounts and transepithelial permeability of PcCLs are 3.24-fold and 7.91-fold higher than that of free insulin with *in vitro* and *in vivo* experiments. Moreover, administration of PcCLs in type 1 diabetic rats performs a prominent hypoglycemic effect and enhances the oral bioavailability up to 11.9%.

### Polymer Nanosphere/Capsule

Relying on the inherent nature of immature dendritic cells to induce immunological tolerance, Silvia et al. (Rodriguez-Fernandez et al., 2018) designed an effective liposome with optimum size and composition that contained phosphatidylserine (existing in the apoptotic cell membrane) and β-cell autoantigens. Acting as apoptotic cell with dynamic typical clearance, phosphatidylserine accelerated the phagocytosis of liposomes and protected dendritic cells viability. The immature dendritic cells then secreted related cytokines, inhibited the proliferation of T cells, reduced the response of antigen-specific T cells to antigens presented by dendritic cells. And then they induced the production of regulatory T cells and re-established immune tolerance to dendritic cells to prevent the further development of T1DM.

**FIGURE 5:**
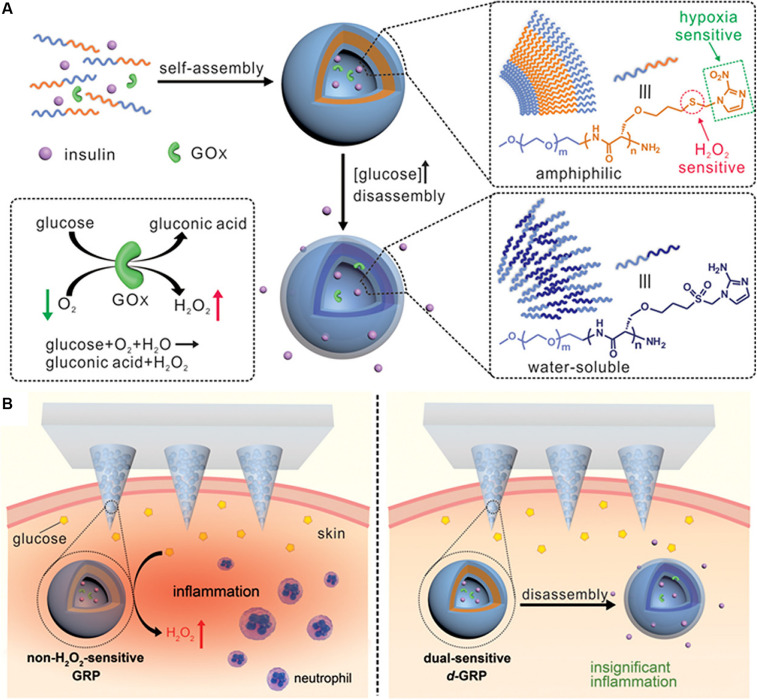
The novel microneedle-array patch for glucose-responsive insulin delivery. Copyright 2017, American Chemical Society. Reprinted from a previous study Yu et al. (2017) with permission. **(A)** Formation and mechanism of hypoxia and H_2_O_2_ dual-sensitive polymersome-based vesicles. **(B)** Schematic of local inflammation caused by non-H_2_O_2_-senstive GRP-loaded microneedle-array patch, and schematic of *d*-GRP-loaded microneedle-array patch for insulin delivery with promising prevention of the side effect associated with inflammation.

As shown in the Figure 5, Yu et al. (2017) exploited a novel painless microneedle-array patch for glucose-responsive insulin delivery, which contained insulin-loaded vesicles self-assembled by hypoxia and H_2_O_2_ double-sensitive diblock copolymers. When blood glucose increases, glucose diffuses through the polymer bilayer membrane to interact with GOx, which produces H_2_O_2_ and local hypoxia in the microenvironment, and then the polymersome-based vesicles dissociate and subsequently release insulin. This system can effectively eliminate the H_2_O_2_ produced by the glucose oxidation, avoid causing tissue damage to the body, enhance the activity of GOx, and improve the glucose responsivity. *In vivo* experiments revealed that this intelligent insulin patch can effectively regulate and control the blood glucose of type 1 diabetic mice for up to 10 h.

Given that the multiple obstacles in the gastrointestinal tract always exist in oral administration and the inspiration of “molecular exchange” between intestinal microbiota and host cells (Li H. et al., 2015), Wu et al. (2017) developed a sort of polyethylene glycol (PEG) nanoparticles incorporated with microbiota metabolite butyrate for oral insulin delivery. Relying on the specific interaction between butyrate and the monocarboxylate transporter (MCT) on cell membranes (Ley et al., 2006), butyrate-dependent cellular uptake was enhanced, and transepithelial transport and intestinal absorption were also obviously improved. Finally, it was suggested that this system induced a stronger hypoglycemic response on diabetic rats and possess a better bioavailability to 9.28%.

**FIGURE 6:**
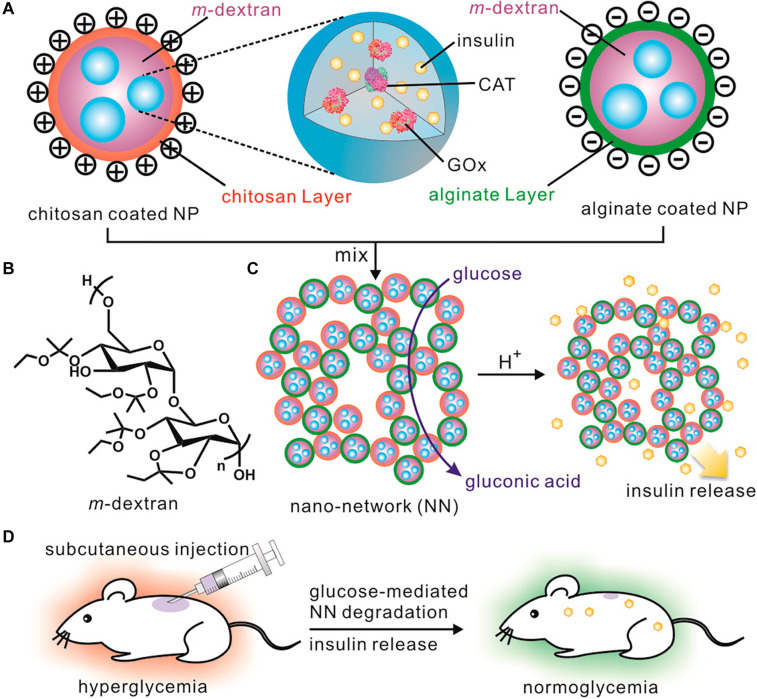
The injectable and acid-degradable polymeric network for glucose-dependent and self-regulated delivery of insulin. Copyright 2013, American Chemical Society. Reprinted from a previous study Gu et al. (2013a) with permission. **(A)** Composition of nanoparticles included in glucose-dependent nano-network (NN). **(B)** Schematic of acidic sensitive acetal-modified dextran. **(C)** The mechanism of nano-network for insulin delivery under hyperglycemic conditions. **(D)** Schematic of glucose-dependent insulin delivery for T1DM treatment using the STZ-induced diabetic mice model.

As shown in Figure 6, an injectable and acid-degradable polymeric network was designed for glucose-dependent and self-regulated delivery of insulin (Gu et al., 2013a). They prepared acetal-modified dextran nanoparticles loaded with recombinant insulin, GOx and CAT, which were then coated with positively charged chitosan and negatively charged sodium alginate using secondary emulsification. This system formed by electrostatic interaction between oppositely charged dextran nanoparticles possessed a stable three-dimensional porous structure. The system can increase the specific surface area of the system and greatly improve the interaction between glucose and its oxidase, which made the system’s glucose faster response. *In vivo* studies confirmed that a single injection of the developed nano-network facilitated stabilization of euglycemia state in mice with T1DM induced by STZ for up to 10 days.

### Polymer Nanogel

As prospective nano carries for drug delivery according to the change of permeability of the polymer membrane, smart nanohydrogels loaded with antidiabetic drugs could be able to rapidly transform their structure-swell or shrink-responding to pH and temperature changes in surrounding media, which are common triggers turning hydrogels from “off” to “on” state. Acting as novel polymeric devices, nanogels could protect protein drugs from enzymatic degradation, delivery them to reach the intestine unmolested, and effectively control the release rate of preloaded drugs (Narayanaswamy and Torchilin, 2019).

Kristy et al. (Wood et al., 2008) designed a class of pH responsive wheat germ agglutinin functionalized composite hydrogels for oral insulin delivery. The nanogel was composed of methacrylic acid (MAA) and PEG (called P (MAA-g-EG). The complexation of the hydrogel was through the temporary physical cross-linking formed by the hydrogen bond between the carboxyl group of MAA and the ether-oxygen of the PEG chain, which made it swelled and dissolved. When reaching the intestinal environment with neutral pH, the carboxyl group of MAA was deprotonated, resulting in ion exclusion between the polymer chains, and the sieve pore size of the hydrogel network increased to release drugs. Therefore, this system could utilize the pH transition between the stomach and the small intestine (from pH 2 to 7) as an environmental trigger to release the drug and deliver it to the target site. At the same time, wheat germ lectin can bind to the mucin in the mucous layer (Gabor et al., 2004), improve the mucosal adhesion characteristics of the carrier and increase the residence time of the carrier at the absorption site, which increase the local concentration of the drugs and improve the bioavailability of the drugs.

**FIGURE 7:**
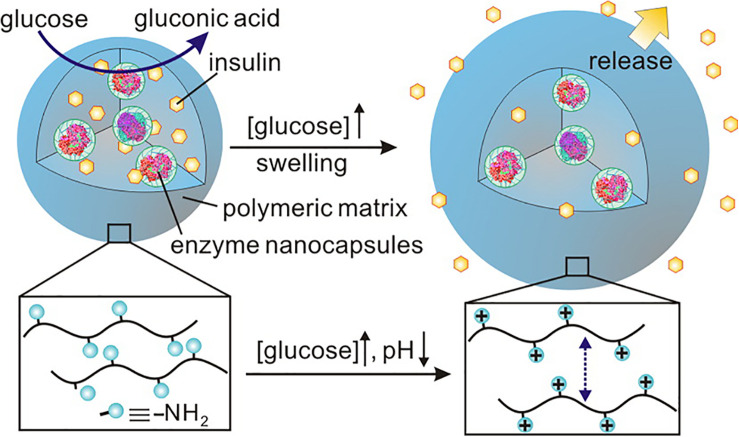
The injectable nanogel with proton sponge effect for closed-loop delivery and release system of insulin. Copyright 2013, American Chemical Society. Reprinted from a previous study Gu et al. (2013b) with permission.

As shown in Figure 7, Gu et al. (2013b) designed uniform injectable nanogel with proton sponge effect for closed-loop delivery and release system of insulin. The nanocapsules loaded with GOx and CAT were encapsulated in the nanogels formed by cross-linking of a pH-responsive chitosan matrix. Under hyperglycemic conditions, a large number of amino groups on the side chain of chitosan are protonated with the formation of gluconic acid. The gel “sponge” exhibited a fivefold volume change due to the electrostatic repulsion, thus releasing the insulin loaded in it.

**FIGURE 8:**
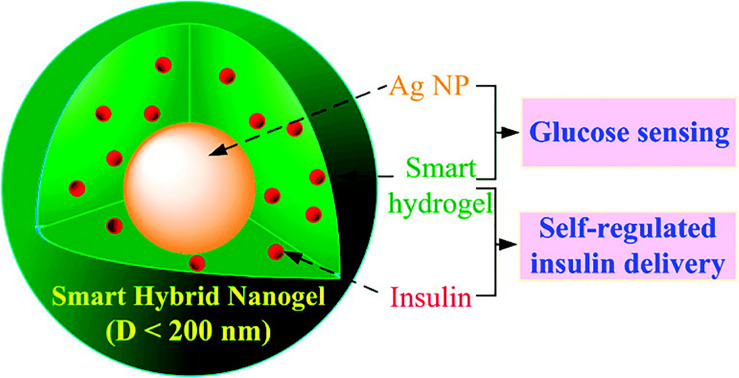
The multifunctional hybrid nanogel for optical detection of glucose and smart-regulated drug delivery. Copyright 2010, American Chemical Society. Reprinted from a previous study Wu et al. (2010) with permission.

As shown in Figure 8, optical detection of glucose, smart-regulated drug delivery, and high drug loading capacity are simultaneously possible using a multifunctional hybrid nanogel (Wu et al., 2010). This delivery system was composed of a copolymer gel shell of poly (4-vinylphenylboronic acid-co-2-(dimethylamino)ethyl acrylate) [p(VPBA-DMAEA)] and Ag NP cores. As a glucose sensing element, p(VPBA-DMAEA) gel shell could swell/shrink in response to the change of glucose concentration with high sensitivity and selectivity, thus controlling the release of preloaded drugs. At the same time, Ag NPs can provide fluorescence signal for hybrid nanogel (Derfus et al., 2004). The swelling/shrinkage of gel shell will affect the fluorescence intensity of Ag NPs. Therefore, the change of glucose concentration can be converted into optical signal for detection and achieving great control of diabetes treatment.

## Conclusion and Prospect

Up to now, owing to the development and advances of drugs therapy, the clinical treatment of diabetes has made significant progress and the condition of patients with diabetes has been well controlled. With the in-depth study of the etiology and the characters of diabetes mellitus, the exploration of novel antidiabetic drugs has been gradually broadened, not only paying great attention to the new targets of non-insulin hypoglycemic drugs and the development and utilization of nano carriers, but also actively looking for treatments that are expected to completely cure diabetes mellitus, including gene therapy and stem cell therapy.

For non-insulin hypoglycemic drugs, new targets such as enzymes and receptors directly related to carbohydrate metabolism and upstream regulatory factors related to energy metabolism have received extensive attention and research. However, given that blood glucose regulation is a complex process of multiple organs, various hormones and diverse signal pathways and the interaction mechanism between targets is not completely clear, great attentions should be paid to the different degrees of adverse reactions that may occur while studying their antidiabetic effect. As the pathogenesis of diabetes mellitus is complicated and more genetic drugs targeting different sites should be developed. Additionally, combination therapy with different kinds of drugs might be a good choice for better therapeutic efficiency.

At present, there are many methods of drug administration for diabetes treatment, such as transdermal delivery, oral delivery, nasal insulin delivery, and pulmonary delivery (Vieira et al., 2019). Among them, oral administration has the most advantage and potential. However, oral drug delivery is faced with many barriers (Reinholz et al., 2018). These barriers mainly include low pH of gastric medium in the stomach, digestive enzymes in the stomach and small intestine, and the mucus and intestinal epithelium. These difficulties seriously limit the bioavailability and therapeutic efficacy of drugs, especially for insulin and genetic drugs. Therefore, it is really important to develop a safe, non-toxic and biodegradable drug delivery systems that can protect drugs from these obstacles and promote their blood absorption. Additionally, overtreatment with antidiabetic drugs may result in hypoglycemia, which can lead to serious side effect such as behavioral and cognitive disturbance, seizures, brain damage, and even worse death. Therefore, it is indispensable to develop more smart delivery systems with appropriate materials and reasonable structures that could achieve controlled and sustained release of drugs in blood after oral administration (Meng et al., 2019). At the same time, the construction of the vehicles should also take into account of the individual differences in pathogenesis, physical fitness and adaptability among patients with diabetes (Zhou et al., 2019).

It has been shown that some organisms with specific structures and function could be utilized to solve the series of barriers in stomach, intestine and blood. For instance, some microbiota metabolites could cross the mucus and intestinal epithelium to blood (Silva et al., 2018). Red blood cells could afford blood circulation up to 120 days because they can avoid the opsonization, immune clearance, and negotiation with vascular systems (Qing et al., 2019). However, their limited source impeded their application. Therefore, bioinspired and biomimetic nanocarriers can be developed for diabetes mellitus treatment. These bioinspired nanocarriers are remarkably potential in resembling the structure and recapitulating one or more functional modules of their native counterparts. They could deliver their cargoes in the way that mimics the natural systems, which is benefit for enhancing blood absorption after oral administration, escaping host immune response, prolonging circulation time, and allowing for actively delivering therapeutic agents to target sites (Chen Z. W. et al., 2019).

As one of the treatment methods that are expected to cure diabetes completely, one main challenges in gene therapy is that fully mature β-cells can not be obtained for insulin replacement therapy. Therefore, the clarification of the gene regulatory mechanism about β-cell differentiation will promote the further development of gene therapy in the field of clinical therapy. In addition, bioprinting approach, as a revolutionary technology, has attracted a lot of attention for the artificial pancreas fabrication in the treatment of T1DM. This technology possesses the advantages of recreating complex morphologies and multicellular environments, and overcoming the limitations of the conventional islet encapsulation technology, such as the hypoxia state, the lack of vascularization, and the diffusion properties of the encapsulation system. At the same time, it could also help pancreatic islets mitigate the autoimmune response and enhance its biological function. With these promising potentials, functional bioprinting could be greatly utilized for better replacement therapy of T1DM (Espona-Noguera et al., 2019).

Therefore, in order to cure diabetes completely, we could start from the gene regulatory mechanism about β-cell differentiation and apoptosis, and design safe and intelligent delivery systems loaded with gene therapeutic drugs. This could help patients with diabetes mellitus get rid of the dependence on exogenous insulin and obtain better compliance. At the same time, we could combine some promising technologies, such as bioinspired nanocarriers and bioprinting for the treatment of diabetes mellitus, which might be a good choice for better therapeutic efficiency.

Above all, the emergence of various drugs and treatments has brought enormous hope for the clinical treatment of diabetes, but how to further clarify the pathogenic mechanism of diabetes and completely cure diabetes is still a serious challenge for researchers.

## Author Contributions

All authors listed have made a substantial, direct and intellectual contribution to the work, and approved it for publication.

## Conflict of Interest

The authors declare that the research was conducted in the absence of any commercial or financial relationships that could be construed as a potential conflict of interest.

## References

[B1] Abdelsalam S. S. Korashy H. M. Zeidan A. Agouni A. (2019). The role of protein tyrosine phosphatase (PTP)-1B in cardiovascular disease and its interplay with insulin resistance. *Biomolecules* 9:286. 10.3390/biom9070286 31319588 PMC6680919

[B2] Ackermann A. M. Wang Z. Schug J. Naji A. Kaestner K. H. (2016). Integration of ATAC-seq and RNA-seq identifies human alpha cell and beta cell signature genes. *Mol. Metab.* 5 233–244. 10.1016/j.molmet.2016.01.002 26977395 PMC4770267

[B3] Ahmad Z. Rasouli M. Azman A. Z. Omar A. R. (2012). Evaluation of insulin expression and secretion in genetically engineered gut K and L-cells. *BMC Biotechnol.* 12:64. 10.1186/1472-6750-12-64 22989329 PMC3469342

[B4] Alberti K. G. Zimmet P. Z. (1998). Definition, diagnosis and classification of diabetes mellitus and its complications. Part 1: diagnosis and classification of diabetes mellitus provisional report of a WHO consultation. *Diabet Med.* 15 539–553. 10.1002/(sici)1096-9136(199807)15:7<539::aid-dia668>3.0.co;2-s 9686693

[B5] Bonner C. Kerr-Conte J. Gmyr V. Queniat G. Moerman E. Thevenet J. (2015). Inhibition of the glucose transporter SGLT2 with dapagliflozin in pancreatic alpha cells triggers glucagon secretion. *Nat. Med.* 21 512–517. 10.1038/nm.3828 25894829

[B6] Bramswig N. C. Kaestner K. H. (2011). Transcriptional regulation of α-cell differentiation. *Diabetes Obes. Metab.* 13(Suppl. 1), 13–20. 10.1111/j.1463-1326.2011.01440.x 21824252

[B7] Bramswig N. C. Kaestner M. F. (2010). Diabetes forum: extreme makeover of pancreatic alpha-cells. *Nature* 464 1132–1133. 10.1038/4641132a 20414295 PMC3982719

[B8] Casellas A. Salavert A. Agudo J. Ayuso E. Jimenez V. Moya M. (2006). Expression of IGF-I in pancreatic islets prevents lymphocytic infiltration and protects mice from type 1 diabetes. *Diabetes Metab. Res. Rev.* 55 3246–3255. 10.2337/db06-0328 17130467

[B9] Chapman K. Holmes M. Seckl J. (2013). 11β -hydroxysteroid dehydrogenases: intracellular gate-keepers of tissue glucocorticoid action. *Physiol. Rev.* 93 1139–1206. 10.1152/physrev.00020.2012 23899562 PMC3962546

[B10] Chen C. Cohrs C. M. Stertmann J. Bozsak R. Speier S. (2017). Human beta cell mass and function in diabetes: recent advances in knowledge and technologies to understand disease pathogenesis. *Mol. Metab.* 6 943–957. 10.1016/j.molmet.2017.06.019 28951820 PMC5605733

[B11] Chen K. F. Jiang S. Hong Y. Li Z. B. Wu Y. L. Wu C. S. (2019). Cationic polymeric nanoformulation: recent advances in material design for CRISPR/Cas9 gene therapy. *Prog. Nat. Sci.* 29 617–627. 10.1016/j.pnsc.2019.10.003

[B12] Chen Z. W. Wang Z. J. Gu Z. (2019). Bioinspired and biomimetic nanomedicines. *Acc. Chem. Res.* 52 1255–1264. 10.1021/acs.accounts.9b00079 30977635 PMC7293770

[B13] Cheung N. Mitchell P. Wong T. Y. (2010). Diabetic retinopathy. *Lancet* 376 124–136. 20580421 10.1016/S0140-6736(09)62124-3

[B14] Cho E. Y. Ryu J. Y. Lee H. A. R. Hong S. H. Park H. S. Hong K. S. (2019). Lecithin nano-liposomal particle as a CRISPR/Cas9 complex delivery system for treating type 2 diabetes. *J. Nanobiotechnol.* 17:19. 10.1186/s12951-019-0452-8 30696428 PMC6350399

[B15] Christiansen E. Hansen S. V. Urban C. Hudson B. D. Wargent E. T. Grundmann M. (2013). Discovery of TUG-770: a highly potent free fatty acid receptor 1 (FFA1/GPR40) agonist for treatment of type 2 diabetes. *ACS Med. Chem. Lett.* 4 441–445. 10.1021/ml4000673 23687558 PMC3654565

[B16] Cichocka E. Wietchy A. Nabrdalik K. Gumprecht J. (2016). Insulin therapy - new directions of research. *Endokrynol. Pol.* 67 314–324. 10.5603/EP.2016.0044 27364374

[B17] Coleman M. A. Jessup C. F. Bridge J. A. Overgaard N. H. Penko D. Walters S. (2016). Antigen-encoding bone marrow terminates islet-directed memory CD8+ T-cell responses to alleviate islet transplant rejection. *Diabetes Metab. Res. Rev.* 65 1328–1340. 10.2337/db15-1418 26961116

[B18] Coughlan K. A. Valentine R. J. Ruderman N. B. Saha A. K. (2014). AMPK activation: a therapeutic target for type 2 diabetes? *Diabetes Metab. Syndr. Obes.* 7 241–253. 10.2147/DMSO.S43731 25018645 PMC4075959

[B19] Davis H. Jones B. V. Dumbadze S. Davis S. N. (2019). Using DPP-4 inhibitors to modulate beta cell function in type 1 diabetes and in the treatment of diabetic kidney disease. *Expert Opin. Investig. Drugs* 28 377–388. 10.1080/13543784.2019.1592156 30848158

[B20] Derfus A. M. Chan W. C. W. Bhatia S. N. (2004). Probing the cytotoxicity of semiconductor quantum dots. *Nano Lett.* 4 11–18. 10.1021/nl0347334 28890669 PMC5588688

[B21] Ehlers M. R. (2016). Immune interventions to preserve β cell function in type 1 diabetes. *J. Investig. Med.* 64 7–13. 10.1097/JIM.0000000000000227 26225763 PMC4732932

[B22] El Naggar N. Kalra S. (2017). Switching from biphasic human insulin to premix insulin analogs: a review of the evidence regarding quality of life and adherence to medication in type 2 diabetes mellitus. *Adv. Ther.* 33 2091–2109. 10.1007/s12325-016-0418-2 27739002

[B23] Espona-Noguera A. Ciriza J. Cañibano-Hernández A. Orive G. Hernández R. M. M. Burgo L. S. D. (2019). Review of advanced hydrogel-based cell encapsulation systems for insulin delivery in type 1 diabetes mellitus. *Pharmaceutics* 11:597. 10.3390/pharmaceutics11110597 31726670 PMC6920807

[B24] Gabor F. Bogner E. Weissenboeck A. Wirth M. (2004). The lectin-cell interaction and its implications to intestinal lectin-mediated drug delivery. *Adv. Drug Deliv. Rev.* 56 459–480. 10.1016/j.addr.2003.10.015 14969753

[B25] Garcia D. Shaw R. J. (2017). AMPK: mechanisms of cellular energy sensing and restoration of metabolic balance. *Mol. Cell.* 66 789–800. 10.1016/j.molcel.2017.05.032 28622524 PMC5553560

[B26] Gentilella R. Pechtner V. Corcos A. Consoli A. (2019). Glucagon-like peptide-1 receptor agonists in type 2 diabetes treatment: are they all the same? *Diabetes Metab. Res. Rev.* 35:e3070. 10.1002/dmrr.3070 30156747

[B27] Gu Z. Aimetti A. A. Wang Q. Dang T. T. Zhang Y. L. Veiseh O. (2013a). Injectable nano-network for glucose-mediated insulin delivery. *ACS Nano* 7 4194–4201. 10.1021/nn400630x 23638642 PMC4107450

[B28] Gu Z. Dang T. T. Ma M. Tang B. B. Cheng H. Jiang S. (2013b). Glucose-responsive microgels integrated with enzyme nanocapsules for closed-loop insulin delivery. *ACS Nano* 7 6758–6766. 10.1021/nn401617u 23834678

[B29] Gupta D. Bhattacharjee O. Mandal D. Sen M. K. Dey D. Dasgupta A. (2019). CRISPR-Cas9 system: a new-fangled dawn in gene editing. *Life Sci.* 232:116636. 10.1016/j.lfs.2019.116636 31295471

[B30] Hollis G. Huber R. (2011). 11β-Hydroxysteroid dehydrogenase type 1 inhibition in type 2 diabetes mellitus. *Diabetes Obes. Metab.* 13 1–6. 10.1111/j.1463-1326.2010.01305.x 21114597

[B31] Hotamisligil G. S. (2006). Inflammation and metabolic disorders. *Nature* 444 860–867. 10.1038/nature05485 17167474

[B32] Ighodaro O. M. (2018). Molecular pathways associated with oxidative stress in diabetes mellitus. *Biomed. Pharmacother.* 108 656–662. 10.1016/j.biopha.2018.09.058 30245465

[B33] Ji Q. (2017). Treatment strategy for type 2 diabetes with obesity: focus on glucagon-like peptide-1 receptor agonists. *Clin. Ther.* 39 1244–1264. 10.1016/j.clinthera.2017.03.013 28526416

[B34] Johnson T. O. Ermolieff J. Jirousek M. R. (2002). Protein tyrosine phosphatase 1B inhibitors for diabetes. *Nat. Rev. Drug Discov.* 1 696–709. 10.1038/nrd895 12209150

[B35] Kaminitz A. Ash S. Askenasy N. (2017). Neutralization versus reinforcement of proinflammatory cytokines to arrest autoimmunity in type 1 diabetes. *Clin. Rev. Allergy Immunol.* 52 460–472. 10.1007/s12016-016-8587-y 27677500

[B36] Katsarou A. Gudbjörnsdottir S. Rawshani A. Dabelea D. Bonifacio E. Anderson B. J. (2017). Type 1 diabetes mellitus. *Nat. Rev. Dis. Primers* 3:17016. 10.1038/nrdp.2017.16 28358037

[B37] Kesharwani P. Gorain B. Low S. Y. Tan S. A. Ling E. C. Lim Y. K. (2018). Nanotechnology based approaches for anti-diabetic drugs delivery. *Diabetes Res. Clin. Pract.* 136 52–77. 10.1016/j.diabres.2017.11.018 29196152

[B38] Klinger S. Poussin C. Debril M. B. Dolci W. Halban P. A. Thorens B. (2008). Increasing GLP-1-induced beta-cell proliferation by silencing the negative regulators of signaling cAMP response element modulator-alpha and DUSP14. *Diabetes Metab. Res. Rev.* 57 584–593. 10.2337/db07-1414 18025410

[B39] Ley R. E. Turnbaugh P. J. Klein S. Gordon J. I. (2006). Microbial ecology: human gut microbes associated with obesity. *Nature* 444 1022–1023. 10.1038/4441022a 17183309

[B40] Li H. Limenitakis J. P. Fuhrer T. Geuking M. B. Lawson M. A. Wyss M. (2015). The outer mucus layer hosts a distinct intestinal microbial niche. *Nat. Commun.* 6:8292. 10.1038/ncomms9292 26392213 PMC4595636

[B41] Li X. H. Wu W. Li J. S. (2015). Glucose-responsive micelles for insulin release. *J. Control. Release* 213 e122–e123. 10.1016/j.jconrel.2015.05.206 27005065

[B42] Mallol C. Casana E. Jimenez V. Casellas A. Haurigot V. Jambrina C. (2017). AAV-mediated pancreatic overexpression of IGF-1 counteracts progression to autoimmune diabetes in mice. *Mol. Metab.* 6 664–680. 10.1016/j.molmet.2017.05.007 28702323 PMC5485311

[B43] Mauricio D. Alonso N. Gratacòs M. (2020). Chronic diabetes complications: the need to move beyond classical concepts. *Trends Endocrinol. Metab.* 31 287–295. 10.1016/j.tem.2020.01.007 32033865

[B44] Meng Q. Y. Hu H. Zhou L. P. Zhang Y. X. Yu B. Shen Y. Q. (2019). Logical design and application of prodrug platforms. *Polymer Chem.* 10, 306–324. 10.1039/c8py01160e

[B45] Moroder L. Musiol H. J. (2017). Insulin-from its discovery to the industrial synthesis of modern insulin analogues. *Angew. Chem. Int. Ed. Engl.* 56 10656–10669. 10.1002/anie.201702493 28548452

[B46] Müller T. D. Finan B. Bloom S. R. Alessio D. D. Drucker D. J. Flatt P. R. (2019). Glucagon-like peptide 1 (GLP-1). *Mol. Metab.* 30 72–130. 10.1016/j.molmet.2019.09.010 31767182 PMC6812410

[B47] Narayanaswamy R. Torchilin V. P. (2019). Hydrogels and their applications in targeted drug delivery. *Molecules* 24:603. 10.3390/molecules24030603 30744011 PMC6384686

[B48] Nespoux J. Vallon V. (2018). SGLT2 inhibition and kidney protection. *Clin. Sci.* 132 1329–1339. 10.1042/CS20171298 29954951 PMC6648703

[B49] Newsholme P. Cruzat V. F. Keane K. N. Carlessi R. de Bittencourt P. I. Jr. (2016). Molecular mechanisms of ROS production and oxidative stress in diabetes. *Biochem. J.* 473 4527–4550. 10.1042/BCJ20160503C 27941030

[B50] Noble J. A. Valdes A. M. (2011). Genetics of the HLA region in the prediction of type 1 diabetes. *Curr. Diab. Rep.* 11 533–542. 10.1007/s11892-011-0223-x 21912932 PMC3233362

[B51] Nyenwe E. A. Kitabchi A. E. (2016). The evolution of diabetic ketoacidosis: an update of its etiology, pathogenesis and management. *Metabolism* 65 507–521. 10.1016/j.metabol.2015.12.007 26975543

[B52] Odegaard J. I. Chawla A. (2013). Pleiotropic actions of insulin resistance and inflammation in metabolic homeostasis. *Science* 339 172–177. 10.1126/science.1230721 23307735 PMC3725457

[B53] Owen C. Lees E. K. Grant L. Zimmer D. J. Mody N. Bence K. K. (2013). Inducible liver-specific knockdown of protein tyrosine phosphatase 1B improves glucose and lipid homeostasis in adult mice. *Diabetologia* 56 2286–2296. 10.1007/s00125-013-2992-z 23832083

[B54] Panzhinskiy E. Hua Y. Culver B. Ren J. Nair S. (2013). Endoplasmic reticulum stress upregulates protein tyrosine phosphatase 1B and impairs glucose uptake in cultured myotubes. *Diabetologia* 56 598–607. 10.1007/s00125-012-2782-z 23178931 PMC3568946

[B55] Piro S. Mascali L. G. Urbano F. Filippello A. Malaguarnera R. Calanna S. (2014). Chronic exposure to GLP-1 increases GLP-1 synthesis and release in a pancreatic alpha cell line (α-TC1): evidence of a direct effect of GLP-1 on pancreatic alpha cells. *PLoS One* 9:e90093. 10.1371/journal.pone.0090093 24587221 PMC3938588

[B56] Poitout V. Lin D. C. (2013). Modulating GPR40: therapeutic promise and potential in diabetes. *Drug Discov. Today* 18 1301–1308. 10.1016/j.drudis.2013.09.003 24051395

[B57] Qian S. Zhang M. He Y. Wang W. Liu S. (2016). Recent advances in the development of protein tyrosine phosphatase 1B inhibitors for Type 2 diabetes. *Future Med. Chem.* 8 1239–1258. 10.4155/fmc-2016-0064 27357615

[B58] Qing X. Zhang Y. T. Zhe L. Hou X. F. Feng N. F. (2019). Red blood cell membrane-camouflaged nanoparticles: a novel drug delivery system for antitumor application. *Acta Pharm. Sin. B* 9 675–689. 10.1016/j.apsb.2019.01.011 31384529 PMC6663920

[B59] Rai V. K. Mishra N. Agrawal A. K. Jain S. Yadav N. P. (2016). Novel drug delivery system: an immense hope for diabetics. *Drug Deliv.* 23 2371–2390. 10.3109/10717544.2014.991001 25544604

[B60] Reinholz J. Landfester K. Mailänder V. (2018). The challenges of oral drug delivery via nanocarriers. *Drug Deliv.* 25 1694–1705. 10.1080/10717544.2018.1501119 30394120 PMC6225504

[B61] Robertson C. C. Rich S. S. (2018). Genetics of type 1 diabetes. *Curr. Opin. Genet. Dev.* 50 7–16. 10.1016/j.gde.2018.01.006 29453110

[B62] Rodriguez-Fernandez S. Pujol-Autonell I. Brianso F. Perna-Barrull D. Cano-Sarabia M. Garcia-Jimeno S. (2018). Phosphatidylserine-liposomes promote tolerogenic features on dendritic cells in human type 1 diabetes by apoptotic mimicry. *Front. Immunol.* 9:253. 10.3389/fimmu.2018.00253 29491866 PMC5817077

[B63] Romer A. I. Sussel L. (2015). Pancreatic islet cell development and regeneration. *Curr. Opin. Endocrinol. Diabetes Obes.* 22 255–264. 10.1097/MED.0000000000000174 26087337 PMC4660868

[B64] Saeedi P. Petersohn I. Salpea P. Malanda B. Karuranga S. Unwin N. (2019). Global and regional diabetes prevalence estimates for 2019 and projections for 2030 and 2045: results from the International Diabetes Federation Diabetes Atlas, 9th edition. *Diabetes Res. Clin. Pract.* 157:107843. 10.1016/j.diabres.2019.107843 31518657

[B65] Senior P. Hramiak I. (2019). Fast-acting insulin aspart and the need for new mealtime insulin analogues in adults with type 1 and type 2 diabetes: a canadian perspective. *Can. J. Diabetes* 43 515–523. 10.1016/j.jcjd.2019.01.004 30872107

[B66] Sheng J. Y. Han L. M. Qin J. Ru G. Li R. X. Wu L. H. (2015). N-trimethyl chitosan chloride-coated PLGA nanoparticles overcoming multiple barriers to oral insulin absorption. *ACS Appl. Mater. Interfaces* 7 15430–15441. 10.1021/acsami.5b03555 26111015

[B67] Shiota C. Prasadan K. Guo P. Ei-Gohary Y. Wiersch J. Xiao X. W. (2013). α-Cells are dispensable in postnatal morphogenesis and maturation of mouse pancreatic islets. *Am. J. Physiol. Endocrinol. Metab.* 305 E1030–E1040. 10.1152/ajpendo.00022.2013 23982158

[B68] Silva J. P. B. Navegantes-Lima K. C. Oliveira A. L. B. Rodrigues D. V. S. Gaspar S. L. F. Monteiro V. V. S. (2018). Protective mechanisms of butyrate on inflammatory bowel disease. *Curr. Pharm. Des.* 24 4154–4166. 10.2174/1381612824666181001153605 30277149

[B69] Smith T. J. (2010). Insulin-like growth factor-I regulation of immune function: a potential therapeutic target in autoimmune diseases? *Pharmacol. Rev.* 62 199–236. 10.1124/pr.109.002469 20392809 PMC2879913

[B70] Song M. (2017). The CRISPR/Cas9 system: their delivery, in vivo and ex vivo applications and clinical development by startups. *Biotechnol. Prog.* 33 1035–1045. 10.1002/btpr.2484 28440027

[B71] Souto E. B. Souto S. B. Campos J. R. Severino P. Pashirova T. N. Zakharova Y. L. (2019). Nanoparticle delivery systems in the treatment of diabetes complications. *Molecules* 24:4209. 10.3390/molecules24234209 31756981 PMC6930606

[B72] Tanaka H. Yoshida S. Minoura H. Negoro K. Shimaya A. Shimokawa T. (2014). Novel GPR40 agonist AS2575959 exhibits glucose metabolism improvement and synergistic effect with sitagliptin on insulin and incretin secretion. *Life Sci.* 94 115–121. 10.1016/j.lfs.2013.11.010 24269216

[B73] Thorel F. Népote V. Avril I. Kohno K. Desgraz R. Chera S. (2010). Conversion of adult pancreatic alpha-cells to beta-cells after extreme beta-cell loss. *Nature* 464 1149–1154. 10.1038/nature08894 20364121 PMC2877635

[B74] Tibaldi J. M. (2014). Evolution of insulin: from human to analog. *Am. J. Med.* 127 S25–S38. 10.1016/j.amjmed.2014.07.005 25282010

[B75] Tudurí E. Bruin J. E. Kieffer T. J. (2012). Restoring insulin production for type 1 diabetes. *J. Diabetes* 4 319–331. 10.1111/j.1753-0407.2012.00196.x 22429761

[B76] Vieira R. Souto S. B. Sánchez-López E. Machado A. L. Severino P. Jose S. (2019). Sugar-lowering drugs for type 2 diabetes mellitus and metabolic syndrome-strategies for in vivo administration: part-II. *J. Clin. Med.* 8:1332. 10.3390/jcm8091332 31466386 PMC6780268

[B77] Wang A. H. Yang T. T. Fan W. W. Yang Y. W. Zhu Q. L. Guo S. (2019). Protein corona liposomes achieve efficient oral insulin delivery by overcoming mucus and epithelial barriers. *Adv. Healthc. Mater.* 8:e1801123. 10.1002/adhm.201801123 30485708

[B78] Wang J. Q. Hu S. Q. Mao W. W. Xiang J. J. Zhou Z. X. Liu X. R. (2019). Assemblies of peptide-cytotoxin conjugates for tumor-homing chemotherapy. *Adv. Functional Mat.* 29 10.1002/adfm.201807446

[B79] Wang X. Zheng P. Huang G. Yang L. Zhou Z. (2018). Dipeptidyl peptidase-4(DPP-4) inhibitors: promising new agents for autoimmune diabetes. *Clin. Exp. Med.* 18 473–480. 10.1007/s10238-018-0519-0 30022375

[B80] Wanner C. Marx N. (2018). SGLT2 inhibitors: the future for treatment of type 2 diabetes mellitus and other chronic diseases. *Diabetologia* 61 2134–2139. 10.1007/s00125-018-4678-z 30132035

[B81] Wong C. Y. Al-Salami H. Dass C. R. (2017). Potential of insulin nanoparticle formulations for oral delivery and diabetes treatment. *J. Control. Release* 264 247–275. 10.1016/j.jconrel.2017.09.003 28887133

[B82] Wong C. Y. Al-Salami H. Dass C. R. (2018a). Microparticles, microcapsules and microspheres: a review of recent developments and prospects for oral delivery of insulin. *Int. J. Pharm.* 537 223–244. 10.1016/j.ijpharm.2017.12.036 29288095

[B83] Wong C. Y. Al-Salami H. Dass C. R. (2018b). Recent advancements in oral administration of insulin-loaded liposomal drug delivery systems for diabetes mellitus. *Int. J. Pharm.* 549 201–217. 10.1016/j.ijpharm.2018.07.041 30071309

[B84] Wood K. M. Stone G. M. Peppas N. A. (2008). Wheat germ agglutinin functionalized complexation hydrogels for oral insulin delivery. *Biomacromolecules* 9 1293–1298. 10.1021/bm701274p 18330990 PMC3071247

[B85] Wu L. Liu M. Shan W. Zhu X. Li L. J. Zhang Z. (2017). Bioinspired butyrate-functionalized nanovehicles for targeted oral delivery of biomacromolecular drugs. *J. Control. Release* 262 273–283. 10.1016/j.jconrel.2017.07.045 28774842

[B86] Wu T. Xie G. X. Ni Y. Liu T. Yang M. Wei H. F. (2015). Serum metabolite signatures of type 2 diabetes mellitus complications. *J. Proteome Res.* 14 447–456. 10.1021/pr500825y 25245142

[B87] Wu W. Mitra N. Yan E. C. Zhou S. (2010). Multifunctional hybrid nanogel for integration of optical glucose sensing and self-regulated insulin release at physiological pH. *ACS Nano* 4 4831–4839. 10.1021/nn1008319 20731458

[B88] Xiao X. Guo P. Shiota C. Zhang T. Coudriet G. M. Fischbach S. (2018). Endogenous reprogramming of alpha cells into beta cells, induced by viral gene therapy, reverses autoimmune diabetes. *Cell Stem Cell* 22 78.e4–90.e4. 10.1016/j.stem.2017.11.020 29304344 PMC5757249

[B89] Xu A. Zhu W. Li T. Li W. Z. Cheng J. Li C. L. (2015). Interleukin-10 gene transfer into insulin-producing β cells protects against diabetes in non-obese diabetic mice. *Mol. Med. Rep.* 12 3881–3889. 10.3892/mmr.2015.3809 25998958

[B90] Yamazaki D. Hitomi H. Nishiyama A. (2018). Hypertension with diabetes mellitus complications. *Hypertens. Res.* 41 147–156. 10.1038/s41440-017-0008-y 29353881

[B91] Yan H. J. Zhu D. C. Zhou Z. X. Liu X. Zhang Z. Liu X. R. (2018). Facile synthesis of semi-library of low charge density cationic polyesters from poly(alkylene maleate)s for efficient local gene delivery. *Biomaterials* 178, 559–569. 10.1016/j.biomaterials.2018.03.050 29653872

[B92] Yeh W. I. Seay H. R. Newby B. Posgai A. L. Moniz F. B. Michels A. (2017). Avidity and bystander suppressive capacity of human regulatory T cells expressing de novo autoreactive T-cell receptors in type 1 diabetes. *Front. Immunol.* 8:1313. 10.3389/fimmu.2017.01313 29123516 PMC5662552

[B93] Yoon J. W. Jun H. S. (2002). Recent advances in insulin gene therapy for type 1 diabetes. *Trends Mol. Med.* 8 62–68. 10.1016/s1471-4914(02)02279-7 11815271

[B94] Yu J. Qian C. Zhang Y. Cui Z. Zhu Y. Shen Q. (2017). Hypoxia and H2O2 dual-sensitive vesicles for enhanced glucose-responsive insulin delivery. *Nano Lett.* 17 733–739. 10.1021/acs.nanolett.6b03848 28079384

[B95] Zhang B. B. Zhou G. Li C. (2009). AMPK: an emerging drug target for diabetes and the metabolic syndrome. *Cell Metab.* 9 407–416. 10.1016/j.cmet.2009.03.012 19416711

[B96] Zhang Y. Jiang G. H. Hong W. J. Gao M. Y. Xu B. Zhu J. Y. (2018). Polymeric microneedles integrated with metformin-loaded and PDA/LA-coated hollow mesoporous sio2 for nir-triggered transdermal delivery on diabetic rats. *ACS Appl. Bio Mater.* 1 1906–1917. 10.1021/acsabm.8b00470 34996291

[B97] Zhang Y. C. Pileggi A. Agarwal A. Molano R. D. Powers M. Brusko T. (2003). Adeno-associated virus-mediated IL-10 gene therapy inhibits diabetes recurrence in syngeneic islet cell transplantation of NOD mice. *Diabetes Metab. Res. Rev.* 52 708–716. 10.2337/diabetes.52.3.708 12606512

[B98] Zhou Q. Shao S. Q. Wang J. Q. Xu C. H. Xiang J. J. Piao Y. (2019). Enzyme-activatable polymer-drug conjugate augments tumour penetration and treatment efficacy. *Nat. Nanotechnol.* 14, 799–809. 10.1038/s41565-019-0485-z 31263194

